# Age- and gender-specific risk of death after first hospitalization for heart failure

**DOI:** 10.1186/1471-2458-10-637

**Published:** 2010-10-22

**Authors:** I Vaartjes, AW Hoes, JB Reitsma, A de Bruin, DE Grobbee, A Mosterd, ML Bots

**Affiliations:** 1 Julius Center for Health Sciences and Primary Care, University Medical Center Utrecht, Utrecht, The Netherlands; 2 Department of Clinical Epidemiology and Biostatistics, Academic Medical Center, Amsterdam, The Netherlands; 3 Statistics Netherlands, The Hague, The Netherlands; 4 Department of Cardiology, University Medical Center Utrecht, Utrecht; 5 Department of Cardiology, Meander Medical Center, Amersfoort, The Netherlands

## Abstract

**Background:**

Hospitalization for heart failure (HF) is associated with high-in-hospital and short- and long-term post discharge mortality. Age and gender are important predictors of mortality in hospitalized HF patients. However, studies assessing short- and long-term risk of death stratified by age and gender are scarce.

**Methods:**

A nationwide cohort was identified (ICD-9 codes 402, 428) and followed through linkage of national registries. The crude 28-day, 1-year and 5-year mortality was computed by age and gender. Cox regression models were used for each period to study sex differences adjusting for potential confounders (age and comorbidities).

**Results:**

14,529 men, mean age 74 ± 11 years and 14,524 women, mean age 78 ± 11 years were identified. Mortality risk after admission for HF increased with age and the risk of death was higher among men than women. Hazard ratio's (men versus women and adjusted for age and co-morbidity) were 1.21 (95%CI 1.14 to 1.28), 1.26 (95% CI 1.21 to 1.31), and 1.28 (95%CI 1.24 to 1.31) for 28 days, 1 year and 5 years mortality, respectively.

**Conclusions:**

This study clearly shows age- and gender differences in short- and long-term risk of death after first hospitalization for HF with men having higher short- and long-term risk of death than women. As our study population includes both men and women from all ages, the estimates we provide maybe a good reflection of 'daily practice' risk of death and therefore be valuable for clinicians and policymakers.

## Background

Heart failure (HF) is a significant and growing health problem, because of the combination of an ageing population and more effective treatment of its major precursor, myocardial infarction [1]. The prevalence of heart failure, a dominant cause of hospitalization in men and women > 65 years in Western population, is estimated to increase from 0.7% for those 45 to 54 years of age to 8.4% for those aged 75 years or older [2].

Current models that are used for the evaluation of the cost-effectiveness and impact on outcomes of heart failure management programmes or predicting the burden on the health care system use overall mortality risks extracted from studies without detailed information on gender- and age-specific mortality or assumptions concerning death rates are made [3-5]. However, age and gender appear to be important predictors of mortality in hospitalized HF patients [6].

Studies presenting short- and long-term mortality risks stratified by age and gender are scarce. Most available studies present the impact of age and gender on mortality using relative risks or hazard ratios, without providing stratified and detailed mortality risks for age and gender subgroups [6-8]. Some studies reported stratified mortality risks, but these were mostly limited to either age or gender [9-12]. Two studies were identified that reported stratified mortality risks by age and gender [13,14]. However, these studies were restricted to short-term risk of death. Only one study could be identified that presented both short- and long-term risk of death stratified by age and gender [10]. However, this study did not include patients from all ages. Therefore, the purpose of the present study was to estimate detailed short- and long-term mortality risks stratified by age and gender in a large nationwide cohort of patients first hospitalized for HF, including both men and women from all ages.

## Methods

For the present study, cohorts were drawn from 1997 and 2000. The choice of these two years was based on pragmatic reasons at the time of the initiation of the project in 2001. The total population of the Netherlands in 1997 and 2000 was 15,567,107 (men 7,696,803, women 7,870,304) and 15,863,950 (men: 7,846,317, women: 8,017, 633), respectively. Approximately 14% of the population was older than 65 years. To construct a cohort of patients admitted for the first time because of HF, information from the national Hospital Discharge Registry (HDR) and the Dutch Population Registry (PR) were linked. Information on cause of death was derived from the Cause of Death Registry of Statistics Netherlands. The registries (which are partly openly available) and linkage procedures have been previously described in detail [15]. In brief, the HDR is a database on admissions, not persons. For each hospital admission a new record is created in the HDR. Following individuals over time based on HDR-information alone is troublesome due to difficulties in identification of different admissions from the same person in time and admissions for the same condition at another hospital (due to referral or to address changes). Yet, linkage (linkage variables date of birth, gender and 4 digits of postal code) with the PR can overcome these issues.

### Study population

All hospital admissions for HF (ICD-9-CM code 402, 428) between January 1^st ^and December 31^st^, 1997 and January 1^st ^and December 31^st^, 2000, were selected from the HDR (which was previously linked with PR). There were 43,738 hospital admissions. In case of multiple HF admissions for an individual within the same year we only used the first admission, yielding a total of 37,386 heart failure patients. Subsequently, information was collected on hospital admission that may have occurred previously (1995-1997 (data earlier than 1995 were not available since linkage with PR is only possible from 1995 onwards) and 1995-2000, respectively) for the same condition. Those with a previous admission for heart failure were excluded (n = 8,333). This resulted in a cohort consisting of 29,053 patients with a first hospitalization for heart failure in 1997 or 2000 in the Netherlands.

### Co-morbidity

The presence of co-morbidity (cardiovascular disease (ICD-9-CM codes 390-459) or diabetes mellitus (ICD-9-CM code 250)) was determined on the basis of the discharge diagnosis of previous hospital admissions or on the basis of a secondary diagnosis at the moment of the index admission. No information on severity of disease, risk factors (hypertension, smoking) or medication use was available in the registry.

### Follow-up

Information on mortality of the patients was obtained by linkage of the cohort with national Cause of Death Register. Linkage of the PR (with which the HDR cohort was previously linked) with the Cause of Death Register (Statistics Netherlands) was performed using a unique identification key and therefore was almost complete. Patients were censored if they migrated out of the Netherlands or if their linkage key was not unique anymore during follow-up. Death was coded using the tenth revision of the International Classification of Disease (ICD-10).

### Data analysis

We analyzed mortality from all causes by examining the proportion of patients that died within 28-days, 1-year, and 5-years after their first admission for heart failure.

Survival time was calculated as the time from the initial admission date in 1997 or 2000 for HF to the date of death from any cause or to the date that a patient was censored, which ever came first. The crude short-term (28 day), 1 year and long-term (5-year) mortality was computed by age and gender according to the actuarial life table method and expressed as percentages. The mortality rate in men was compared to mortality rate in women by calculating relative risks (with 95% CI). Cox regression models were used for each period to study differences between men and women in their risk of death with and without adjusting for potential confounders (age and previous admissions for cardiovascular disease or diabetes mellitus). Data were analyzed with SPSS software, version 14.0 (SPSS Inc, Chicago, Illinois, USA). All analyses were performed in agreement with privacy legislation in the Netherlands [16].

## Results

A total of 29,053 patients (mean age 76 ± 11) with a first hospitalization for congestive heart failure in 1997 or 2000 were identified. General characteristics are provided in Table 1. Eighteen, 38% and 67% of all men and 27%, 36% and 66% of all women died within 28 days, 1-year and 5 years, respectively.

**Table 1:** Characteristics of patients with a hospital admission for heart failure in 1997 or 2000.

	Men	Women	Total
Number of patients	14,529	14,524	29,053

Age at admission (years)			
Mean	74	78	76
Standard deviation	11	11	11

Prior co-morbidities (%)			
cardiovascular disease	44.2	37.5	40.9
- ischemic heart disease	21.0	15.0	18.0
- *acute myocardial infarction*	*9.8*	*7.5*	*8.6*
- stroke	3.2	3.3	3.2
- peripheral arterial disease	7.9	4.6	6.2
- other cardiovascular disease	24.7	22.5	23.6
diabetes mellitus	15.1	20.4	17.8

Type of hospital (%)			
- academic	13.0	9.9	11.5

Length of stay (days)	12	13	12

Origin (%)			
-native Dutch	90	89	89

### Cause of death

Cardiovascular disease was the most frequent cause of death at 28-days, 1-year and 5-year (table 2). The contribution of cardiovascular diseases as cause of death decreased with increasing follow-up time while the contribution of cancer as a cause of death increased.

**Table 2:** Causes of death of patients during follow-up after hospital admission for heart failure in the Netherlands.

	28 days		1-year		5-years	
**Cause of death**	**Men (n = 2,644)**	**Women (n = 2,661)**	**Men (n = 5,455)**	**Women (n = 5,273)**	**Men (n = 9,677)**	**Women (n = 9,558)**

Co-morbidities (%)						
Cardiovascular diseases	65.2	70.2	62.8	65.9	59.2	60.6
- ischemic heart disease	29.9	24.8	28.2	22.3	26.3	20.0
- *AMI*	*16.6*	*14.9*	*14.5*	*13.0*	*13.1*	*11.1*
- congestive heart failure	17.0	21.4	14.7	18.0	12.6	15.7
- stroke	2.3	3.2	3.0	4.3	3.7	5.3
- peripheral arterial diseases	1.9	1.2	2.7	1.9	3.2	2.2
- other cardiovascular diseases	14.1	19.6	14.1	19.3	13.5	17.7
						
Cancer	7.4	4.8	9.8	6.7	11.2	7.5
- lung cancer	2.2	0.6	3.3	0.7	3.6	0.8
						
Diseases of respiratory system	14.8	10.5	13.1	9.2	13.0	10.0
- COPD	7.3	4.6	6.9	4.3	6.6	4.4
Complications from DM	2.5	3.6	3.2	4.8	3.8	5.5

AMI: acute myocardial infarction, COPD: chronic obstructive respiratory disease, DM: diabetes mellitus

### 28 day mortality

Short term mortality risk increased with age in men and women (from 7.5% in men younger than 55 years to 32.9% in men older than 85 years and from 6.9% in women younger than 55 years to 27.2% in women older than 85 years). Higher mortality rates in men compared to women were found across all ages above 65 years (Table 3). Crude overall mortality was similar for men and women (hazard ratio 0.99; 95% CI 0.94 to 1.05). However, after adjustment for potential confounders (age, previous admission for cardiovascular diseases or diabetes mellitus) mortality was higher in men than in women (hazard ratio 1.21; 95% CI 1.14 to 1.28) (Table 4).

**Table 3:** Mortality risk at 28 days, 1 year and 5 years after first hospital admission (1997 or 2000) for heart failure in the Netherlands, by age and gender.

	No. of men	No. of women	Age	Men		Women		RR (95% CI) for Men vs Women
					
				No. of Deaths	Percentage Deaths	No. of Deaths	Percentage Deaths	
28-day mortality	814	525	<55	61	7.5	36	6.9	1.09 (0.73-1.63)
	672	303	55-59	47	7.0	16	5.3	1.32 (0.76-2.30)
	1,118	595	60-64	111	9.9	54	9.1	1.09 (0.80-1.49)
	1,851	1,107	65-69	195	10.5	116	10.5	1.28 (1.10-1.48)
	2,597	1,826	70-74	419	16.1	231	12.7	1.13 (1.01-1.27)
	3,058	2,835	75-79	538	17.6	440	15.5	1.29 (1.17-1.41)
	2,579	3,208	80-84	667	25.9	645	20.1	1.21 (1.11-1.31)
	1,840	4,125	85+	606	32.9	1,123	27.2	1.21 (1.11-1.31)

1-year mortality	814	525	<55	140	17.2	78	14.9	1.16 (0.90-1.49)
	672	303	55-59	124	18.5	51	16.8	1.10 (0.82-1.48)
	1,118	595	60-64	245	21.9	128	21.5	1.02 (0.84-1.23)
	1,851	1,107	65-69	507	27.4	245	22.1	1.24 (1.08-1.41)
	2,597	1,826	70-74	892	34.3	518	28.4	1.21 (1.11-1.33)
	3,058	2,835	75-79	1,205	39.4	940	33.2	1.19 (1.11-1.33)
	2,579	3,208	80-84	1,264	49.0	1,255	39.1	1.25 (1.18-1.33)
	1,840	4,125	85+	1,078	58.6	2,058	49.9	1.17 (1.12-1.23)

5-year mortality	814	525	<55	278	34.2	145	27.6	1.24 (1.05-1.46)
	672	303	55-59	273	40.6	102	33.7	1.21 (1.01-1.45)
	1,118	595	60-64	521	46.6	250	42.0	1.11 (0.99-1.24)
	1,851	1,107	65-69	1,028	55.5	510	46.1	1.21 (1.12-1.30)
	2,597	1,826	70-74	1,691	65.1	1,000	54.8	1.19 (1.13-1.25)
	3,058	2,835	75-79	2,195	71.8	1,765	62.3	1.15 (1.11-1.20)
	2,579	3,208	80-84	2,089	81.0	2,315	72.2	1.12 (1.09-1.15)
	1,840	4,125	85+	1,602	87.1	3,471	84.1	1.03 (1.01-1.06)

**Table 4:** Gender differences in short- and long-term mortality after a first hospital admission (1997 or 2000) for heart failure in the Netherlands

	Heart failure
	
	HR (95% CI)*
Crude	
28 days	0.99 (0.94-1.05)
1 year	1.04 (1.00-1.08)
5 years	1.03 (1.00-1.06)
	
Adjusted**	
28 days	1.21 (1.14-1.28)
1 year	1.26 (1.21-1.31)
5 years	1.28 (1.24-1.31)

*Hazard ratio (95% Confidence interval) men vs women.

**adjusted for age and prior co-morbidities (previous admissions for cardiovascular disease or diabetes mellitus)

### One-year mortality

One-year mortality risk increased with age in men and women (from 17.2% in men younger than 55 years to 58.6% in men older than 85 years and from 14.9% in women younger than 55 years to 49.9% in women older than 85 years). One-year mortality was higher in men than in women across all ages above 65 years (Table 3). The crude overall mortality was similar for men and women at 1 year (hazard ratio 1.04; 95% CI 1.00 to 1.08). However, after adjustment for potential confounders mortality was higher for men compared to women (Table 4).

### Five-year mortality

Five-year mortality risk increased with age in men and women (from 34.2% in men younger than 55 years to 87.1% in men older than 85 years and from 27.6% in women younger than 55 years to 84.1% in women older than 85 years). A higher mortality in men compared to women was found across all ages; but this difference was not statistically significant for men and women between 60 and 64 years (Table 3). After adjustment for potential confounders overall five-year mortality was higher for men compared to women (Table 4).

## Discussion

The present study using a nationwide cohort of 29,053 patients first hospitalized for heart failure shows clear age- and gender differences in short- and long-term mortality risk.

We describe a high mortality over a follow-up of 5 years with men having a higher short- and long-term risk of death than women.

Overall mortality risks are presented in several population based cohort studies and in intervention studies evaluating the effect of drug treatment in HF. Similar overall short- and long-term mortality risks were presented in population based cohorts [17,18]. The ARIC study [19] however, reported lower short- and long-term mortality risk. Though patients were considerable younger in the ARIC study (mean age 57 years versus 76 years). Trials [20-23] also report lower mortality risks (1-year mortality risks ranged from 8 to 31% compared to 37%). This likely reflects the younger age and less co-morbidity of patients participating in trials evaluating the effect of drug treatment for HF [24-26].

The limited available age- and gender specific data show that the Swedish short-term mortality risks were lower, the Scottish short- and long-term mortality risks were higher [10], whereas in the United States 30-day mortality risks were lower while 1-year mortality risks were similar [13]. The differences in mortality risk between the countries might reflect differences in health care system and treatment as well as differences in patient characteristics. The latter however remains a matter of speculation since we had no information about the medication prescribed and limited information on clinical characteristics of the patients.

The higher mortality risks in men compared to women reported in this study have also been reported in previous large epidemiological studies [27]. A better long-term survival after 10-15 years has been reported in women across all age groups [28]. In contrast to these studies, survival was higher in men than in women in the SOLVD trial [29]. In studies of patients admitted to hospital, women were found to have a lower [13,30] or equal mortality [9]. Possible explanations for the association between gender and mortality risk that have been reported include that men are more likely than women to have an ischemic etiology of their heart failure and that ventricular ejection fraction is higher in female than in male heart failure patients with a nonischemic cause [30].

Our results provide insight in age-and gender-specific risk of death following initial admission for heart failure. As our study population includes both men and women from all ages, the estimates we provide maybe a good reflection of 'daily practice' risk of death and therefore be valuable for clinicians and policymakers. Furthermore, these results can be used for the evaluation of the cost-effectiveness and impact on outcomes of heart failure management programmes that focuses on secondary prevention.

The strength of our study is the large size of the cohort obtained from usual care with a large age range and information on both men and women. Even though the validity of national registries has been questioned, several studies have shown that for the Netherlands, the validity is adequate [31]. Positive predictive values for the use of ICD-9 code 428 to identify patients with HF varies between 80.0% [32] and 94.3% [33]. The quality of the applied linkage strategy has been excellent [31]. The cause of death information used in our study was not validated by medical records or autopsy. As a result, the degree of misclassification in the cause of death is unquantifiable. However, as in almost every study using data from vital statistics, some degree of misclassification is inevitable. In addition, the validity of the Dutch national Cause of Death registry has been reported to be higher than the average validity of eight countries in the European Community [34].

To appreciate the findings of this study the following issues needs consideration. The search for previous admissions to maximum of 6 years before, might have led to that some "first" heart failure patients were actually recurrent heart failure patients. The risk of recurrence is the highest within the first two years (44% readmission within 6 months [35] and 70% readmission within 2 years after a first admission for heart failure [36]). The inclusion of recurrent heart failure patients may cause overestimation of absolute mortality rates (as recurrent heart failure patients may be more severe who are likely to have a higher mortality risk) but its extent is difficult to quantify.

## Conclusions

In conclusion, this study clearly shows age- and gender differences in short- and long-term risk of death after first hospitalization for heart failure with men having a higher short- and long-term risk of death than women. As our study population includes both men and women from all ages, the estimates we provide maybe a good reflection of 'daily practice' risk of death and therefore be valuable for clinicians and policymakers.

## Competing interests

The authors declare that they have no competing interests.

## Authors' contributions

IV performed the statistical analysis and drafted the manuscript. AH conceived of the study and commented the draft. JR participated in the design of this study and commented the draft. AB participated in the design of this study and commented the draft. DG conceived of the study and commented the draft. AM conceived of the study and commented the draft. MB conceived of the study, and participated in its design and coordination. All authors read and approved the final manuscript.

## Pre-publication history

The pre-publication history for this paper can be accessed here:

http://www.biomedcentral.com/1471-2458/10/637/prepub
